# In Situ Growth of Ni-MOF Nanorods Array on Ti_3_C_2_T_x_ Nanosheets for Supercapacitive Electrodes

**DOI:** 10.3390/nano13030610

**Published:** 2023-02-03

**Authors:** Shengzhao Li, Yingyi Wang, Yue Li, Jiaqiang Xu, Tie Li, Ting Zhang

**Affiliations:** 1 NEST Lab, Department of Chemistry, College of Sciences, Shanghai University, Shanghai 200444, China; 2 i-Lab, Nano-X Vacuum Interconnected Workstation, Key Laboratory of Multifunction Nanomaterials and Smart Systems, Suzhou Institute of Nano-Tech and Nano-Bionics (SINANO), Chinese Academy of Sciences (CAS), 398 Ruoshui Road, Suzhou 215123, China; 3 Gusu Lab for Advanced Materials, Suzhou 215123, China

**Keywords:** MXene, MOFs, in situ growth, nanorods, supercapacitor

## Abstract

For the energy supply of smart and portable equipment, high performance supercapacitor electrode materials are drawing more and more concerns. Conductive Ni-MOF is a class of materials with higher conductivity compared with traditional MOFs, but it continues to lack stability. Specifically, MXene (Ti_3_C_2_T_x_) has been employed as an electrochemical substrate for its high mechanical stability and abundant active sites, which can be combined with MOFs to improve its electrochemical performance. In this paper, a novel Ni-MOF nanorods array/Ti_3_C_2_T_x_ nanocomposite was prepared via a facile hydrothermal reaction, which makes good use of the advantages of conductive Ni-MOF and high strength Ti_3_C_2_T_x_. The high density forest-like Ni-MOF array in situ grown on the surface of Ti_3_C_2_T_x_ can provide abundant active electrochemical sites and construct a pathway for effective ion transport. The formation of a “Ti-O···Ni” bond accomplished during an in situ growth reaction endows the strong interfacial interaction between Ni-MOF and Ti_3_C_2_T_x_. As a result, the Ni-MOF/Ti_3_C_2_T_x_ nanocomposite can achieve a high specific capacitance of 497.6 F·g^−1^ at 0.5 A·g^−1^ and remain over 66% of the initial capacitance when the current density increases five times. In addition, the influence of the Ti_3_C_2_T_x_ concentration and reaction time on the morphology and performance of the resultant products were also investigated, leading to a good understanding of the formation process of the nanocomposite and the electrochemical mechanism for a supercapacitive reaction.

## 1. Introduction

Energy supplement devices with ultrathin volume, high energy density, and long lifespan are arousing amounts of interest along with the miniaturization and portability of smart equipment [1,2,3]. Among them, high performance supercapacitors have propelled rapidly because of their fast charge/discharge speed, convenient assembly, and high-level safety regardless of if the miniaturization process is performed [4,5,6]. However, further progress is severely constrained by their low energy density. Thus, it is necessary to investigate novel electrode materials with superior conductivity, high specific surface area, and easy preparation to overcome this restriction [7,8,9].

Metal-organic frameworks (MOFs) have been widely focused on in the past decades because of their considerable specific surface area, tunable structural architectures, and abundant active sites [10,11,12]. Formerly, the low conductivity limits traditional MOFs’ appearance in a number of current advanced technologies with the requirement of high electrical conductivity. With the appearance of conductive MOFs such as M-HHTP (M is Co, Ni, Cu, etc. and HHTP is hexahydroxytriphenylene) [13,14], they gradually exhibit greater potential in supercapacitors [15,16]. For instance, Mircea et al. reported the first example of a supercapacitor electrode made entirely from neat conductive MOFs as active materials [17]. However, the improved conductivity is still restricted by the shortages of low specific capacitances and structural stability in the long-term cycling process. Therefore, other conductive matrixes including conductive polymers (e.g., polypyrrole or polyaniline) and carbon-based materials (e.g., graphene or carbon nanotubes) have been brought into compositing with MOF to guarantee the effective control on electrochemical performances [18,19,20,21,22,23].

M_n+1_X_n_T_x_ (*n* = 1~4), in which M represents transition metals (e.g., Ti, V, etc.), X is C or/and N, and T_x_ stands for terminal groups, such as -O, -OH, and -F, on the surface [24,25], and also named MXene, has been developed quickly since it has excellent conductivity [26,27], many surface groups for functional modification [28,29], and other fascinating tunable properties [30,31]. Recently, Ti_3_C_2_T_x_, the most widely studied MXene, has been used in supercapacitors by diversiform-processing techniques [32,33,34]. Nevertheless, there are also shortcomings in the easy stacking and oxidization of Ti_3_C_2_T_x_ nanosheets during the electrochemical reaction process, which can be remitted by modifying them with other active materials [35,36].

Herein, combined with the above merits of MOFs, it can be clearly suggested that the nanocomposite synthesized from Ti_3_C_2_T_x_ and conductive Ni-MOF will effectively make the utmost of their advantages since MXenes improve composite conductivity and provide sufficient surface groups to interact with MOFs while the Ni-MOF plays an important role in offering plentiful active sites and large specific surface area, reasonably obtaining incremental attention in electrochemical applications [37,38,39]. However, the direct combination out of in situ growth provides weak interfacial bonding, which can result in high transport impedance and low cyclic stability. In addition, the relationship between structural architectures and electrochemical performances is also unrevealed through regulating reaction conditions. Briefly, there is a significant attempt to develop MXene-MOF nanocomposites to solve the above issues on the account of the in situ growth strategy.

In this study, we propose a strategy for the in situ growing of forest-like hexagonal-shaped Ni-MOF (Ni-HHTP) directly on the surface of Ti_3_C_2_T_x_ rather than the simple mixture. The advantages of Ti_3_C_2_T_x_ and Ni-MOF are effectively combined in this nanocomposite to address the aforementioned shortcomings, enabling reversible transport of electrons and ions at the interface as well as preventing the collapse of porous MOF structures during the insertion and de-intercalation of electrolyte ions. Additionally, the in situ growth mechanism of this nanocomposite was studied through analyzing the morphology and structure of serial products prepared from the controlled factors of reactant concentration and reaction time. Moreover, the electrochemical characterizations reveal that the Ni-MOF/Ti_3_C_2_T_x_ nanocomposite exhibits a high specific capacitance value of 497.6 F·g^−1^ (0.5 A·g^−1^) with a high cyclic stability of 85% during 1000 cycles, which can be due to the synergistic effect of the underlying MXene substrate and porous MOF architectures. Furthermore, an all-solid-state asymmetric supercapacitor was also demonstrated by exploiting the Ni-MOF/Ti_3_C_2_T_x_ nanocomposite as a positive-electrode material, successful in lighting a LED. The obtained results will possess great potential for electrochemical devices.

## 2. Materials and Methods

### 2.1. Synthesis of MXene Ti_3_C_2_T_x_

As the most reported method [40], Ti_3_C_2_T_x_ nanosheet was synthesized as follows: 8 g of LiF was added to 100 mL of 9 M HCl in a PTFE bottle as the etchant. A total of 5 g Ti_3_AlC_2_ was slowly added to the above etchant in an ice bath. The reaction was heated under 40 °C and magnetic stirring was maintained for 24 h. A dark green Ti_3_C_2_T_x_ slurry was formed after washing by deionization water via centrifugation at 3500 rpm for 5 min until the pH reached 5~6. The Ti_3_C_2_T_x_ powder was collected through centrifugating the slurry at 12,000 rpm for 30 min and drying the sediment at 80 °C in a vacuum oven for 24 h.

### 2.2. Synthesis of Ni-MOF/Ti_3_C_2_T_x_

The Ni-MOF/Ti_3_C_2_T_x_ nanocomposite was synthesized as follows: 0.5 mg/mL of Ti_3_C_2_T_x_ solution was prepared under ultrasonication for 30 min. An amount of 40 mg Ni(OAc)_2_∙4H_2_O and 28 mg HHTP was dispersed in 4 mL deionized water, respectively, before mixing the two homogeneous solutions above. Then, 8 mL mixture was added to 8 mL Ti_3_C_2_T_x_ solution in a 20 mL vessel. The reaction was heated in an oven at 80 °C for 8 h and then cooled down to room temperature within 3 h. Black sediment was collected at the bottom of the vessel. The Ni-MOF/Ti_3_C_2_T_x_ sample was obtained by washing the sediment using deionized water and ethanol for several times. The length of Ni-MOF nanorods in situ grown on the Ti_3_C_2_T_x_ nanosheet was efficiently controlled by the hydrothermal reaction time. The pure Ni-MOF powder was synthesized through the same procedure without Ti_3_C_2_T_x_.

### 2.3. Activation of Ni-MOF/Ti_3_C_2_T_x_

The Ni-MOF/Ti_3_C_2_T_x_ powder was activated after immersing it into deionized water at 80 °C overnight and exchanging the solvent with acetone that was replaced everyday for three days. The acetone-exchanged Ni-MOF/Ti_3_C_2_T_x_ powder was collected after drying at 80 °C in a vacuum oven for 24 h to remove guest molecules.

### 2.4. Fabrication of the Electrodes in a Traditional Three-Electrode System

Electrochemical performance was conducted by a CHI 660E workstation in a three-electrode system. A platinum wire electrode and a Hg/HgO electrode was used as the counter electrode and reference electrode, respectively. A concentration of 6.0 M KOH was chosen as the electrolyte. The working electrode was prepared by the mixing active materials, acetylene black and polytetrafluoroethylene (PTFE), with a weight ratio of 8:1:1, and then coating the mixture above on a 1 cm × 3 cm nickel foam. N-methyl-2-pyrrolidone was chosen as the additive when mixing and the coating area was about 1 cm^2^. Then, a thin foil was achieved by pressing the nickel foam under 10.0 MPa. The mass-loading of the active material was about 3–5 mg.

### 2.5. Fabrication of the Asymmetric Supercapacitor

The Ni-MOF/Ti_3_C_2_T_x_ nanocomposite and the active carbon were used as the positive and negative electrode materials, respectively. The mass ratio of electrode materials is determined by C^+^m^+^ = C^−^m^−^, where C^+^ and C^−^ are the specific capacitance of Ni-MOF/Ti_3_C_2_T_x_ and active carbon. The KOH/PVA solution was used as the electrolyte and then sandwiched by the electrode. Lastly, the flexible supercapacitor was packeted with PET membrane. The resulting electrochemical measurement was conducted via a CHI 660E workstation.

## 3. Results and Discussion

### 3.1. Sample Preparation and Structure Characterization

**Figure 1:**
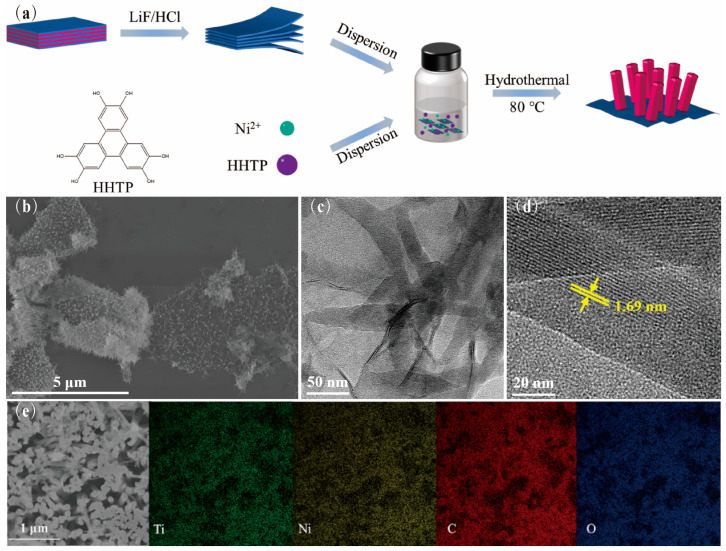
(**a**) Schematic illustration of the synthesis of Ni-MOF/Ti_3_C_2_T_x_ sample; (**b**) SEM image of Ni-MOF/Ti_3_C_2_T_x_; (**c**) TEM image of Ni-MOF/Ti_3_C_2_T_x_; (**d**) HRTEM image of Ni-MOF nanorods; and (**e**) EDS mapping of Ni-MOF/Ti_3_C_2_T_x_.

The schematic diagram of the synthesis procedure of the Ni-MOF/Ti_3_C_2_T_x_ nanocomposite is depicted in Figure 1a. Initially, the Ti_3_C_2_T_x_ solution was prepared from its corresponding nanosheet powder that was obtained through etching the Al layers using LiF/HCl and dried in a vacuum; the two-dimensional Ti_3_C_2_T_x_ nanosheets’ morphology is shown in Figure S1. Next, a homogeneous precursor mixture was obtained by adding Ni(OAc)_2_∙4H_2_O and HHTP into the above Ti_3_C_2_T_x_ solution, which was subsequently put in an 80 °C oven for a hydrothermal reaction. Lastly, the expected Ni-MOF/Ti_3_C_2_T_x_ sample was received via washing the collected reaction sediment with DI water and ethyl alcohol repeatedly. Afterwards, the controlled products prepared from the various reactant concentrations and hydrothermal times were employed to investigate the in situ growth mechanism and electrochemical performance of the nanocomposite.

The morphology of the as-prepared Ni-MOF/Ti_3_C_2_T_x_ nanocomposite (0.5 mg∙mL^−1^ and 12 h) was revealed using the images from scanning electron microscopy (SEM), as shown in Figure 1b. It can be seen that the regular forest-like Ni-MOF nanorod arrays with hexagonal architectures and lengths of up to 550 nm were grown in situ on the surface of Ti_3_C_2_T_x_, vertically, forming strong interfacial bonds, which can bring about low electrochemical impedance and high cyclic stability for this well-designed nanocomposite. In addition, there was no obvious stacking phenomenon of the 2D Ti_3_C_2_T_x_ nanosheets since the in situ growth of the Ni-MOF arrays can also effectively prevent the agglomeration of lamellae that occurred in the hydrothermal process. Furthermore, the elemental mapping of this Ni-MOF/Ti_3_C_2_T_x_ nanocomposite given by the energy dispersive spectrometer (EDS) also demonstrates the uniform in situ growth of the Ni-MOF arrays arranged on the surface of the Ti_3_C_2_T_x_ nanosheets, as consistent with the above SEM results (Figure 1e). This leads to abundant active sites for redox reactions and effective transport pathways for electron transfer and ion diffusion, which makes a great contribution to enhance the electrochemical properties of this novel Ni-MOF/Ti_3_C_2_T_x_ nanocomposite.

Furthermore, the nature of the crystal structure of the as-synthesized Ni-MOF/Ti_3_C_2_T_x_ products was characterized by transmission electron microscopy (TEM) (Figure 1c). It can be evidently seen in Figure 1c that the nanocomposite consists of numerous Ni-MOF nanorods in situ forming on the surface of the 2D Ti_3_C_2_T_x_ nanosheets, which is in agreement with the above SEM observations. Additionally, the apparent wrinkles of the 2D nanosheets and the whole forest-like constructions implicitly exhibit the Ni-MOF/Ti_3_C_2_T_x_ sample endowed with steady and strong interfacial bonding because there is no obvious crack or collapse occurring even after ultrasonication treatment. The high resolution TEM (HRTEM) (Figure 1d) reveals that the characteristic lattice fringe of 1.69 nm belongs to the (100) crystal face of the Ni-MOF structure.

**Figure 2:**
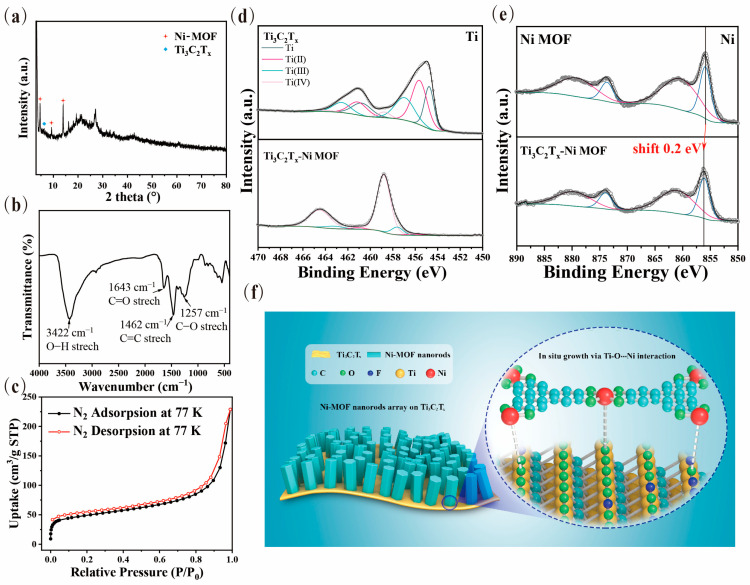
(**a**) XRD pattern, (**b**) FT-IR spectrum, and (**c**) BET curve of the Ni-MOF/Ti_3_C_2_T_x_ sample; (**d**,**e**) XPS peaks of Ti and Ni elements in Ni-MOF/Ti_3_C_2_T_x_, respectively; and (**f**) the illustration of Ti-O···Ni interaction between Ni-MOF and Ti_3_C_2_T_x_.

As shown in Figure 2a, according to previous studies, the characteristic diffraction peaks of the Ni-MOF/Ti_3_C_2_T_x_ nanocomposites at 2θ of 4.8°, 9.7°, and 14.2° are attributed to the (100), (200), and (210) lattice planes of Ni-MOF [41], respectively, suggesting a high crystallinity of the products. Furthermore, compared with the peaks of the mixture of Ni-MOF and Ti_3_C_2_T (Figure S1), the location of the (002) plane shifts from 6.6° to approximately 6.2° and the intensity decreases to near disappearance, demonstrating that the (100) face of Ni-MOF nanorods were grown in situ on the (002) face of the Ti_3_C_2_T_x_ nanosheets rather than simply mixture [42]. It can also be inferred that there was no stacking of Ti_3_C_2_T_x_ nanosheets from the pattern in which more electrolyte anions can be contained and serve as electroactive species in the Faradaic redox reaction.

Fourier transform-infrared (FT-IR) measurements were performed to confirm the interactions between the components of Ti_3_C_2_T_x_ and Ni-MOF based on the location changes of the functional groups in the Ni-MOF/Ti_3_C_2_T_x_ nanocomposite. As shown in Figure 2b, the spectrum of nanocomposite exhibits the typical absorption features of its above components containing the stretching mode of the O-H group at 3422 cm^−1^, the C=O group at 1643 cm^−1^, graphene at 1462 cm^−1^, and the C-O group at 1257 cm^−1^_,_ which can be observed from Ti_3_C_2_T_x_ and Ni-MOF in Figure S2 [41]. It is known that the O-H stretching vibration peak can evidently appear red-shift when the interactions take place from the molecular level. It is noted in Figure S2 that the O-H stretching vibration peak at 3422 cm^−1^ for the Ni-MOF/Ti_3_C_2_T_x_ sample shows a certain red-shift compared with that at 3441 cm^−1^ for Ti_3_C_2_T_x_, indicating there exists strong interactions between Ni-MOF and Ti_3_C_2_T_x_, as consistent with the XRD results.

As exhibited in Figure 2c, the Brunauer–Emment–Teller (BET) test of the Ni-MOF/Ti_3_C_2_T_x_ nanocomposite gains a specific surface area of 167.74 m^2^∙g^−1^ and an average pore width of 14.3 nm. A type I isotherm profile demonstrates its microporous nature. All of these confirm its relatively large specific surface area by virtue of the well-defined forest-like nanorod arrays with a hierarchically hexagonal configuration, which is beneficial for enhancing the electrochemical properties.

Furthermore, X-ray photoelectron spectroscopy (XPS) was performed to illustrate the specific interaction sites of the above FT-IR analysis via characterizing the changes of the composition and the valence state of the elements between the Ni-MOF, Ti_3_C_2_T_x_, and Ni-MOF/Ti_3_C_2_T_x_ nanocomposites. The fitting spectra of Ti 2p, Ni 2p, and O 1s of the Ni-MOF/Ti_3_C_2_T_x_ sample were detected, which demonstrate a high degree of interaction achieved between Ti_3_C_2_T_x_ and Ni-MOF (Figure S4). As shown in Figure 2e, the Ni 2p_3/2_ and 2p_1/2_ peaks of the nanocomposite (855.9 eV and 873.6 eV, respectively) show a definite change (0.2 eV shifting) compared with those of Ni-MOF (856.1 eV and 873.8 eV, respectively), revealing the formation of the “Ti-O···Ni” interaction between Ni-MOF and Ti_3_C_2_T_x_ as similar to the formation mechanism of “V-O···Ni”, as previously reported [39]. Furthermore, the Ti 2p spectra provide fairly powerful evidence to confirm the in situ growth mechanism of the nanocomposite as depicted in Figure 2d. It can be evidently found that the three doublets of the Ti 2p spectra of the Ni-MOF/Ti_3_C_2_T_x_ products present a majority of Ti^4+^ (458.8/464.4 eV) and few of Ti^2+^ (456.1/461.1 eV) and Ti^3+^ (457.6/463.2 eV), which are much different from the eight deconvoluted peaks of Ti (454.7 eV/460.7 eV), Ti^2+^ (455.6 eV461.1 eV), Ti^3+^ (456.7 eV/462.5 eV), and Ti^4+^ (458.8 eV/464.4 eV) of the pure Ti_3_C_2_T_x_ nanosheets. In addition, the Ti^4+^ in the nanocomposite turns out not to be in the form of TiO_2_, which can be proved by no obvious TiO_2_ characteristic peak in the XRD spectra. As a consequence, this is a sign that an abundance of active Ti^4+^ sites can be formed during hydrothermal reactions, as previously reported [43,44], and they play an important role in the in situ anchoring of Ni-MOF via the “Ti-O···Ni” interaction. The spectra of O 1s are shown in Figure S5 and demonstrates further evidence that the strong “Ti-O···Ni” interaction occurs between Ti_3_C_2_T_x_ and Ni-MOF during the in situ growth process. In other words, the XPS results correspond well with the conclusions of XRD and FT-IR, which ensures that the “Ti-O···Ni” interaction forms during the in situ growth of Ni-MOF via anchoring at active Ti sites on the surface of Ti_3_C_2_T_x_, as illustrated in Figure 2f.

### 3.2. Morphology Controlled by Concentration and Reaction Time

**Figure 3:**
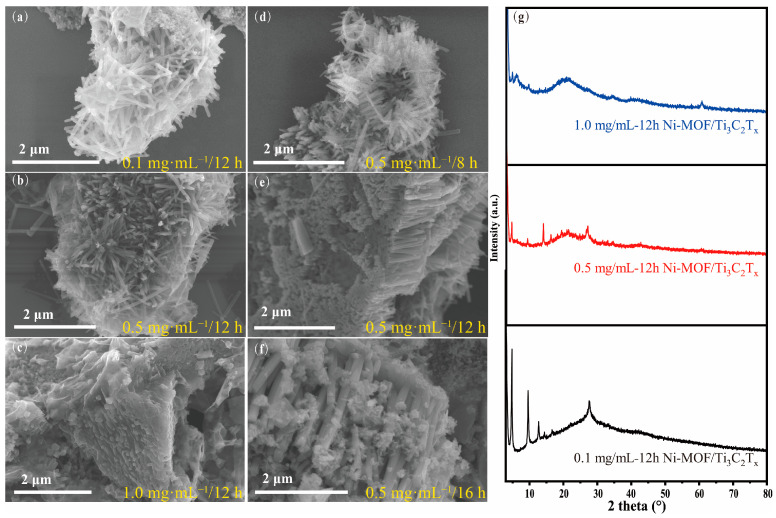
(**a**–**c**) SEM images of the Ni-MOF/Ti_3_C_2_T_x_ samples within the same 12 h reaction time at different Ti_3_C_2_T_x_ concentrations of 0.1 mg·mL^−1^, 0.5 mg·mL^−1^, and 1.0 mg·mL^−1^, respectively; (**d**–**f**) SEM images of the Ni-MOF/Ti_3_C_2_T_x_ products at the same concentration 0.5 mg·mL^−1^ of Ti_3_C_2_T_x_ within different reaction times of 8 h, 12 h, and 16 h, respectively; and (**g**) XRD pattern of the Ni-MOF/Ti_3_C_2_T_x_ samples grown at different Ti_3_C_2_T_x_ concentrations.

The concentration of Ti_3_C_2_T_x_ and the reaction time have a direct effect on the micromorphology and structure of the Ni-MOF/Ti_3_C_2_T_x_ products, which makes an important impact on the electrochemical performance. On the one hand, the concentration of the Ti_3_C_2_T_x_ solution determines whether the forest-like Ni-MOF array vertically grows on the surface of Ti_3_C_2_T_x_ successfully. When the concentration of the solution is 0.1 mg∙mL^−1^, a small quantity of Ti_3_C_2_T_x_ is entirely covered by the relatively excessive Ni-MOF nanorods in no particular order (Figure 3a), resulting in the intensity of the corresponding (002) peaks of Ti_3_C_2_T_x_ disappearing in its XRD patten as shown in the black line of Figure 3g. When the solution increases to 1.0 mg∙mL^−1^, compared with the morphology of the sample obtained at 0.5 mg∙mL^−1^ (Figure 3c), Ni-MOF displays a nanosheet structure with a low density of distribution on the surface of Ti_3_C_2_T_x_. As a result, the XRD pattern shows the characteristic peaks of Ti_3_C_2_T_x_ and Ni-MOF at 6.1° and 4.8°, respectively, indicating that Ni-MOF adhered to the surface prevents Ti_3_C_2_T_x_ from accumulating and increases the lattice space of the Ti_3_C_2_T_x_ nanosheets (blue line in Figure 2g). In addition, the intensity of Ti_3_C_2_T_x_’s and Ni-MOF’s characteristic peaks all show strong signals because the low density of Ni-MOF cannot cover the surface and leads to exposure of the (002) face of the Ti_3_C_2_T_x_ rather than disappearance or weakness of the intensity as for the above two samples. On the other hand, the reaction time decides the size of the final Ni-MOF products. It can be clearly found that the length of Ni-MOF increases from ~250 nm of 8 h, ~550 nm of 12 h, to ~2.5 μm of 18 h by comparing the results of Figure 3d–f. To summarize, the concentration of Ti_3_C_2_T_x_ and the reaction time play pivotal roles in controlling the architecture of the Ni-MOF/Ti_3_C_2_T_x_ nanocomposite, which is due to providing different growth environments for Ni-MOF. A low Ti_3_C_2_T_x_ concentration and a limited reaction time provide insufficient growth conditions for Ni-MOF anchoring on the surface of Ti_3_C_2_T_x_. By contrast, when up to 1.0 mg∙mL^−1^, the quantity of the Ni ions and the HHTP ligand cannot satisfy continuous growth after anchoring. Under 0.5 mg∙mL^−1^ and for 12 h, the Ni-MOF nanorods can grow uniformly on the surface of the Ti_3_C_2_T_x_ nanosheets. The different morphologies significantly impact the electrochemical performance of the products.

### 3.3. Electrochemical Properties

**Figure 4:**
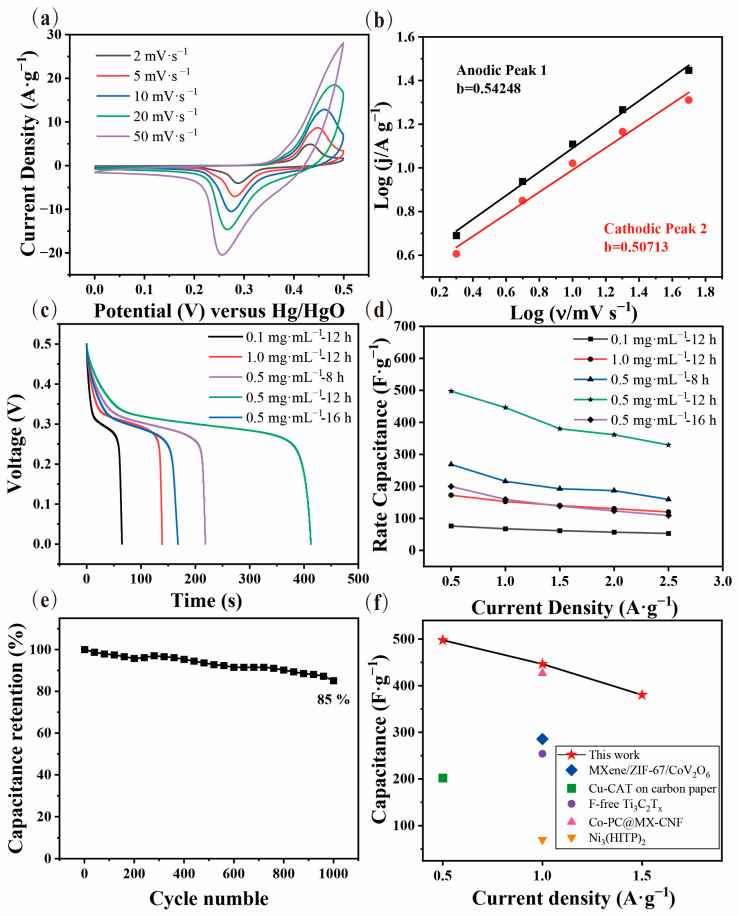
(**a**) CV curves of the Ni-MOF/Ti_3_C_2_T_x_ sample prepared by growth condition of 0.5 mg∙mL^−1^ and 12 h; (**b**) the corresponding determination of *b-values* based on cathodic and anodic peaks; (**c**) GCD curves of the Ni-MOF/Ti_3_C_2_T_x_ samples obtained by different growth conditions; (**d**) the comparation of rate capacitance between Ni-MOF/Ti_3_C_2_T_x_ products with different growth conditions; (**e**) cycling behaviors of the Ni-MOF/Ti_3_C_2_T_x_ sample of 0.5 mg∙mL^−1^ and 12 h growth conditions; and (**f**) ragone plots of the Ni-MOF/Ti_3_C_2_T_x_ sample compared with previous studies [17,38,47,48,49].

The cyclic voltammetry (CV) and galvanostatic charge–discharge (GCD) tests were performed to measure the electrochemical capacitance via the traditional three-electrode system using 6 M KOH as the electrolyte and Hg/HgO as the reference electrode. The redox peak can be clearly seen from the CV curves rather than rectangular shapes, which demonstrates the typical pseudocapacitive behavior (Figure 4a). As shown in Figure 4c, the GCD curves clearly show that the specific capacitance of the Ni-MOF/Ti_3_C_2_T_x_ nanocomposite can reach as high as 497.6 F·g^−1^ (0.5 A·g^−1^), which can remain over 66% of the initial capacitance when the current density increases five times from 0.5 to 2.5 A·g^−1^. Additionally, it shows a long discharge plateau period, which has great potential in practical applications. The good electrochemical performance of the Ni-MOF-Ti_3_C_2_T_x_ nanocomposite can be attributed to the synergetic combination of the excellent conductivity of the Ti_3_C_2_T_x_ substrate and the abundant redox active sites of MOF for electron transfer and ion transport. Because of the strong interaction formed during in situ growth, the Ni-MOF/Ti_3_C_2_T_x_ nanocomposite can maintain over 85% of the initial capacitance after 1000 cycles (Figure 4e). The large capacitance mainly displays a pseudocapacitance behavior due to the abundant active metal sites on the surface of the 2D Ti_3_C_2_T_x_ nanosheets, the porous skeleton (electric double layer capacitor), and the benzoquinone reaction that happened at the skeleton of the Ni-MOF, which can provide a good capacity for charge storage [45,46]. Additionally, the change of the impedance shows an obvious change (Figure S10), which might be caused by the less irreversible passivation behavior of the nanomaterials during the electrochemical cycles.

Furthermore, it can be explicitly proved that the architectures of the Ni-MOF/Ti_3_C_2_T_x_ samples prepared from different parameters have an important effect on the electrochemical capacities, as shown in the GCD curves of Figure 4c. When the concentration of the Ti_3_C_2_T_x_ solution is 0.1 and 1.0 mg∙mL^−1^, the capacitance is 76.5 and 172.5 F·g^−1^, respectively, because no order structure hinders ion transport and the failure of the forest-like Ni-MOF nanorods cannot provide sufficient active sites and pathways. In addition, the Ni-MOF/Ti_3_C_2_T_x_ samples prepared by 8 h and 16 h have much lower electrochemical capacitances of 268.8 F·g^−1^ and 200.0 F·g^−1^ at 0.5 A·g^−1^, respectively. It can be deduced that there is a trade-off effect between the number of redox active sites and the length of the ion pathways. When the growth time is short (8 h) or the Ti_3_C_2_T_x_ substrate is insufficient (0.1 mg∙mL^−1^), there are not abundant sites for an electrochemical reaction. Pathways that are too long are formed from the Ni-MOF nanorods (16 h, 1.0 mg∙mL^−1^) and can also impede the available movement of ions, resulting in an optimal Ni-MOF/Ti_3_C_2_T_x_ sample obtained from a growth time of 12 h and a Ti_3_C_2_T_x_ solution of 0.5 mg∙mL^−1^ with larger capacitance and capacitance retention, as revealed in Figure 4d. Additionally, the comparison of the rate capacitance and the energy density, as shown in Figure 4d, Figures S7 and S8, exhibits the Ni-MOF/Ti_3_C_2_T_x_ nanocomposite possesses an excellent energy density of 223.9 KWh·kg^−1^ at a power density of 1953.3 W·kg^−1^ (Figure S9), which surpasses the purely conducive MOF and Ti_3_C_2_T_x_ and some of the MOF-Ti_3_C_2_T_x_ composites.

**Figure 5:**
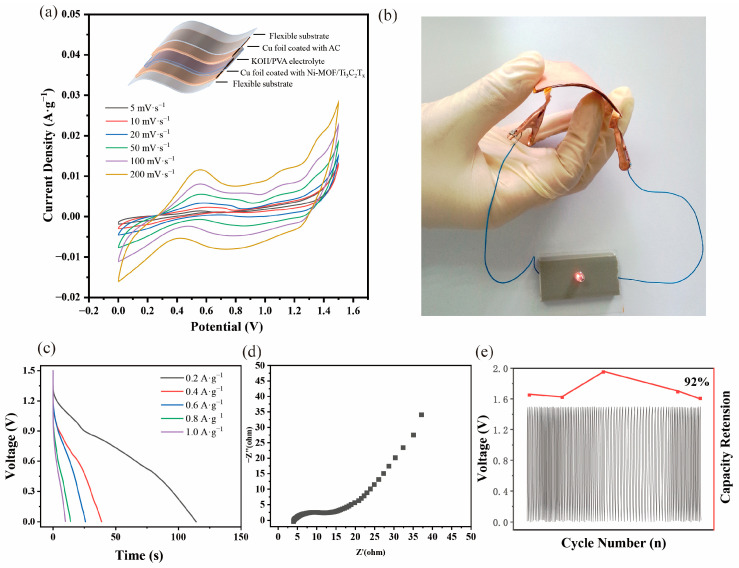
(**a**) Schematic illustration of flexible supercapacitor and the CV electrochemical test of the device; (**b**) success in lighting a LED; (**c**) the GCD curves of the device; (**d**) the EIS curves of the device; and (**e**) the cycle test of the device.

Lastly, as depicted in Figure 5a, an all-solid-state asymmetric flexible supercapacitor was assembled by employing a Ni-MOF/Ti_3_C_2_T_x_ nanocomposite as the cathode and activated carbon as the anode. Additionally, the voltage window was fixed to 1.5 V by the cycle voltammetry test on the anode and cathode, respectively (Figure S11). The flexible device exhibits a specific capacitance of 128 F·g^−1^, calculated through CV tests, and the devices have succeeded in lighting a LED as shown in Figure 5b. The GCD test of the device shows a good charge and discharge curve as depicted in Figure 5c. Additionally, the EIS test (Figure 5d) shows the small impedance of the flexible supercapacitor, which can reduce the energy loss effectively. Moreover, the device shows good electrochemical stability of more than 90 GCD cycles, as shown in Figure 5e. Additionally, it can be observed from Figure S12, with the bending angle change from 0° to 30° and 60°, the CV curves show an inconspicuous change, which also demonstrates their good stability when applied to the bending scenario.

## 4. Conclusions

In summary, we have developed a novel Ni-MOF/Ti_3_C_2_T_x_ electrode material with a high order nanorods forest in situ grown on 2D Ti_3_C_2_T_x_ nanosheets. By controlling the Ti_3_C_2_T_x_ concentration and the hydrothermal reaction time, the relationship between the microstructure and electrochemical performance is successfully revealed. Benefiting from Ni-MOF’s abundant active redox sites and Ti_3_C_2_T_x_’s high conductivity, the in situ combination identifies an electrochemical performance of 497.6 F·g^−1^ (0.5 A·g^−1^) and a capacitive retention of over 66% of the initial capacity when the current density is increased five times, which can be attributed to the synergistic effect of Ni-MOF and Ti_3_C_2_T_x_. The “Ti-O···Ni” bond formed during in situ growth offers a strong interfacial combination between Ni-MOF and Ti_3_C_2_T_x_. Ni-MOF provides sufficient active sites, effective ion transportation pathways, and avoids stacking of Ti_3_C_2_T_x_, while Ti_3_C_2_T_x_ acts as a high conductivity material. The device shows good performance and stability in tests, which shows its potential in practical applications. Thus, this work provides a new material for electrochemical applications.

## Data Availability

The data presented in this study are available within the article.

## Supplementary Materials

The following supporting information can be downloaded at: https://www.mdpi.com/article/10.3390/nano13030610/s1, Figure S1. The SEM image of the Ti_3_C_2_T_x_ nanosheets, Figure S2. The XRD pattern of the Ti_3_C_2_T_x_, Figure S3. FT-IR pattern of (a) Ti_3_C_2_T_x_ and (b) Ni-MOF, respectively, Figure S4. The pore size distribution of Ni-MOF/Ti_3_C_2_T_x_, Figure S5. XPS spectra of Ni-MOF/Ti_3_C_2_T_x_, Figure S6. High-resolution O 1s spectra of Ti_3_C_2_T_x_, Ni-MOF, Ni-MOF/Ti_3_C_2_T_x_, respectively, Figure S7. Comparation of rate capacitance of Ni-MOF/Ti_3_C_2_T_x_, with different growth conditions, Figure S8. Comparation of energy density of Ni-MOF/Ti_3_C_2_T_x_, with different growth conditions, Figure S9. Energy density at different Power density of Ni-MOF/Ti_3_C_2_T_x_, Figure S10. The EIS test of the Ni-MOF/Ti_3_C_2_T_x_ before and after stability test, Figure S11. Energy density at different Power density of Ni-MOF/Ti_3_C_2_T_x_, Figure S12. The CV test under original or bending states.
