# Supplementary material for: In Situ Growth of Ni-MOF Nanorods Array on Ti3C2Tx Nanosheets for Supercapacitive Electrodes
Source: Nanomaterials (Basel). 2023 Feb 3;13(3):610. doi: 10.3390/nano13030610 (PMC9921429; doi:10.3390/nano13030610)
Supplement: Supplementary file 1 [file nanomaterials-13-00610-s001.zip › nanomaterials-2166263-supplementary.pdf]

In situ growth of Ni-MOF nanorods array on $Ti_3C_2T_x$ nanosheets for electrodes

Shengzhao Li[a, b], Yingyi Wang[b], Yue Li[b], Jiaqiang Xu[*,a], Tie Li[*,b, c], Ting Zhang[*,b]

[a]NEST Lab, Department of Chemistry, College of Sciences, Shanghai University, Shanghai 200444, China

[b]i-Lab, Nano-X Vacuum Interconnected Workstation, Key Laboratory of Multifunction Nanomaterials and Smart Systems, Suzhou Institute of Nano-Tech and Nano-Bionics (SINANO), Chinese Academy of Sciences (CAS), 398 Ruoshui Road, Suzhou, 215123, P. R. China.

[c]Gusu Lab for Advanced Materials, Suzhou, 215123, P. R. China

1. Methods for calculation of specific capacitance

CV test

The gravimetric capacitances were calculated according to the following equations:

$$Cg=\frac{\int IdV}{2vm\Delta V}$$

Where C_g (F g^{-1}) is the gravimetric capacitance of the electrode, I (A) is the charge-discharge current, v(V/s) is the scan rate, m(g) is the mass of the working electrode. ΔV (V) is voltage window.

GCD test

The volumetric capacitances were calculated according to the following equations:

$$Cg=\frac{2i_m\int Vdt}{V^2}$$

$$E=1/2\cdot CgV^2$$

$$P=E/t$$

Where Cg (F g^{-1}) is the gravimetric capacitance of the electrode, i_m (A) is the charge-discharge current density, Δt (s) is the discharge time, V (V) represents voltage drop on discharging, E (Wh·kg^{-1})is the energy density of the electrode, P (W·kg^{-1})is the power density of the electrode.

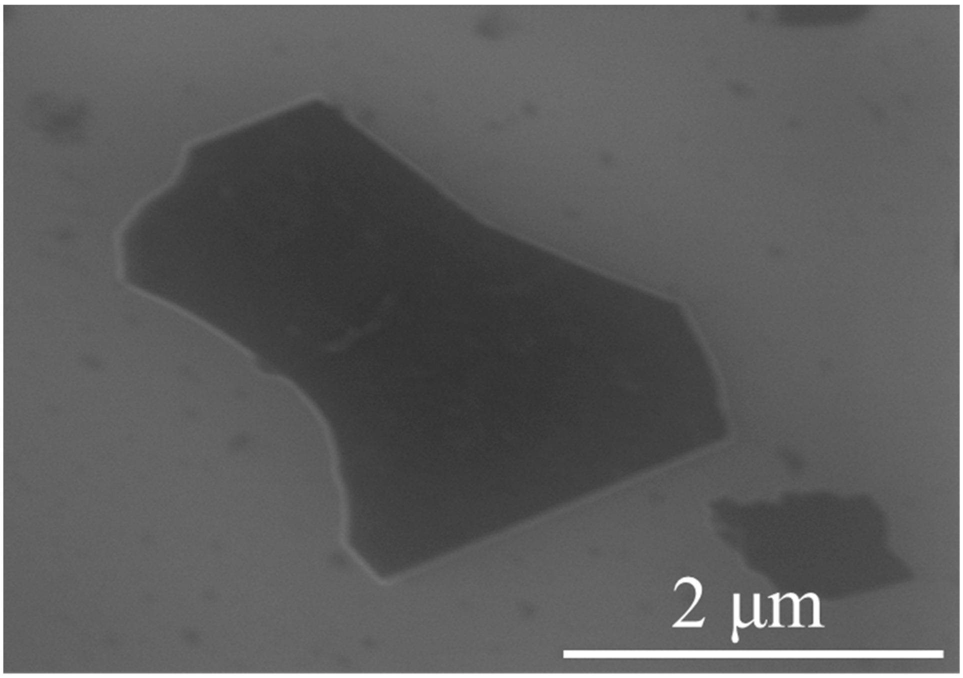

Figure S1. The SEM image of the $Ti_3C_2T_x$ nanosheets.

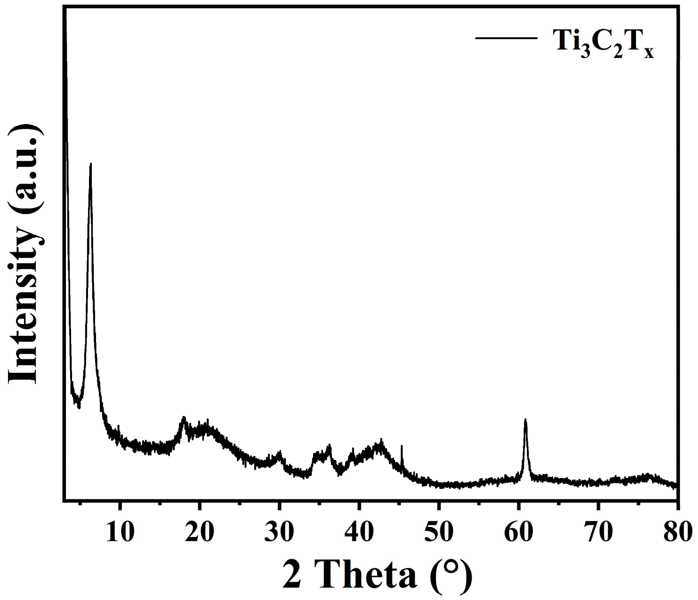

Figure S2. The XRD pattern of the $Ti_3C_2T_x$.

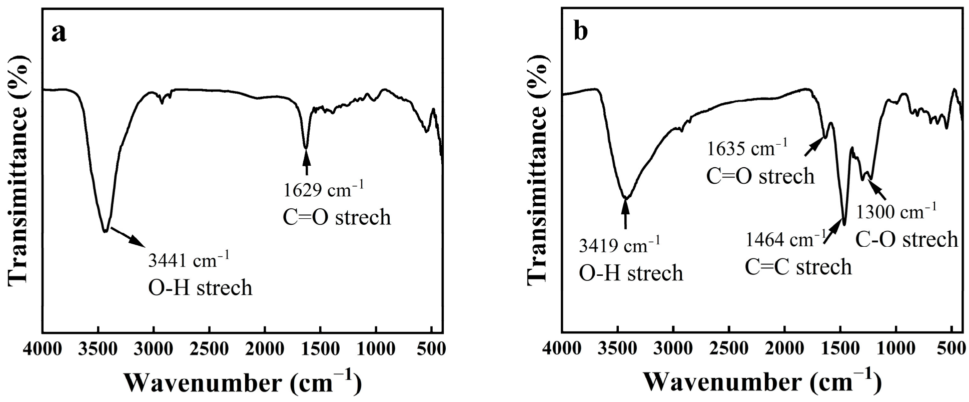

Figure S3. FT-IR pattern of (a) $Ti_3C_2T_x$ and (b) Ni-MOF, respectively.

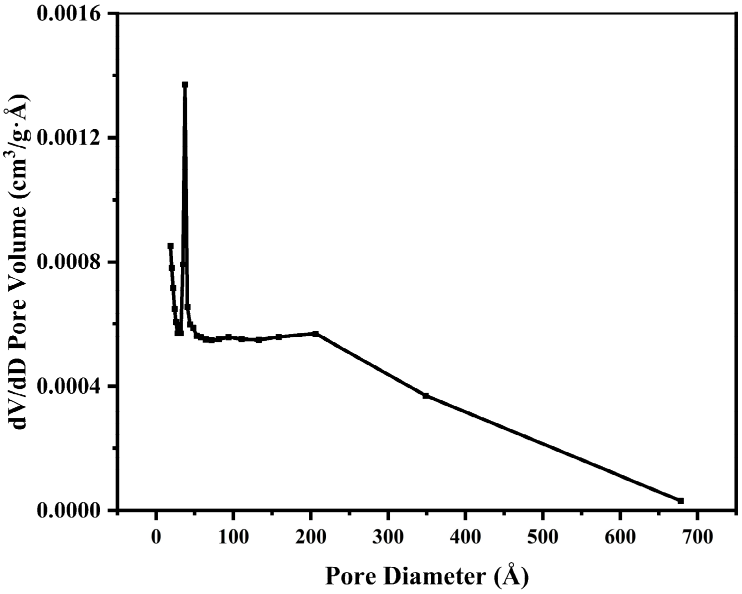

Figure S4. The pore size distribution of Ni-MOF/$Ti_3C_2T_x$.

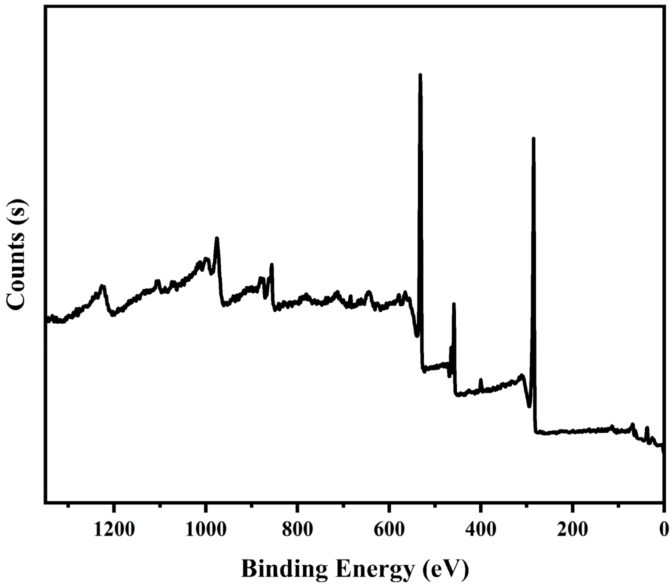

Figure S5. XPS spectra of Ni-MOF/$Ti_3C_2T_x$.

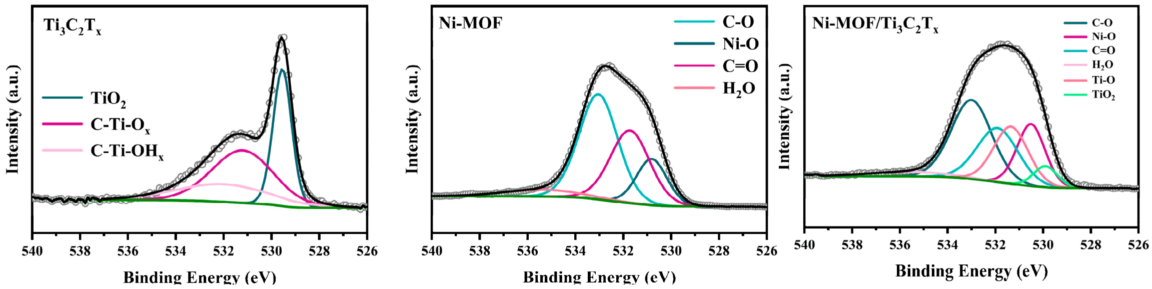

Figure S6. High-resolution O 1s spectra of $Ti_3C_2T_x$, Ni-MOF, Ni-MOF/$Ti_3C_2T_x$, respectively.

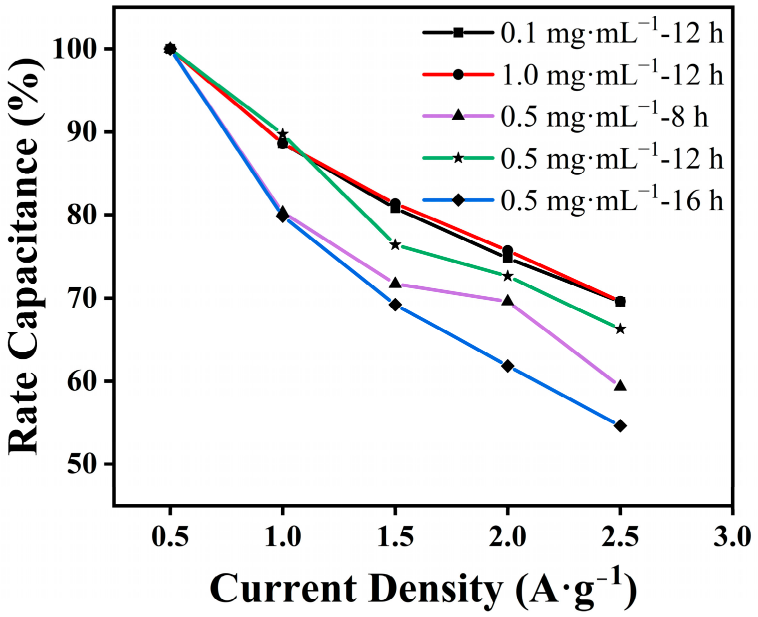

Figure S7. Comparation of rate capacitance of Ni-MOF/$Ti_3C_2T_x$, with different growth conditions.

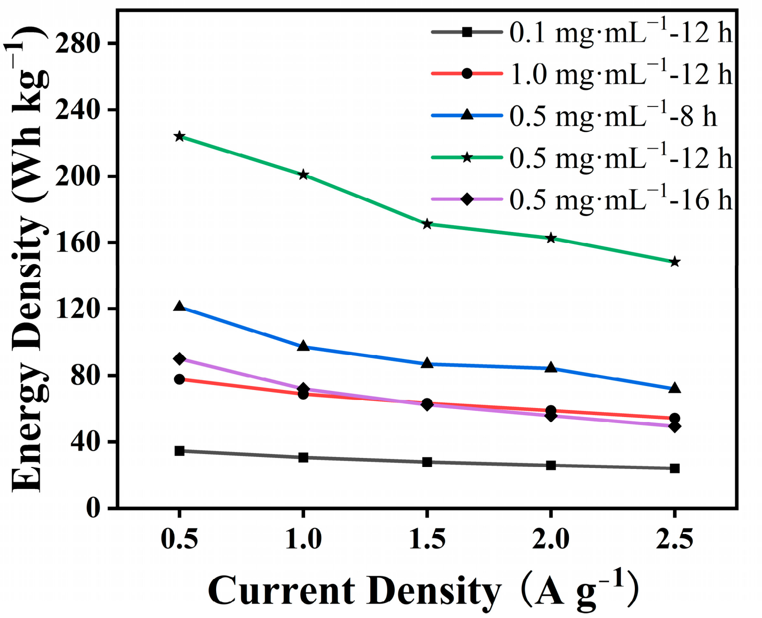

Figure S8. Comparation of energy density of Ni-MOF/$Ti_3C_2T_x$, with different growth conditions.

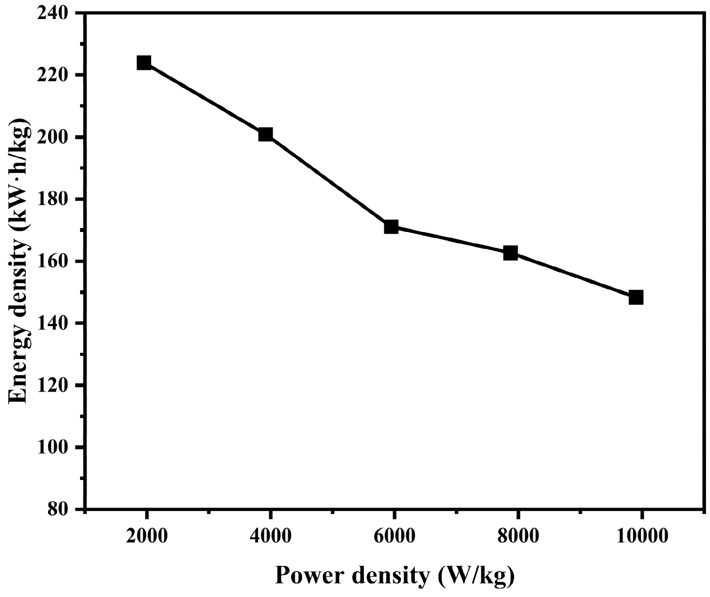

Figure S9. Energy density at different Power density of Ni-MOF/$Ti_3C_2T_x$.

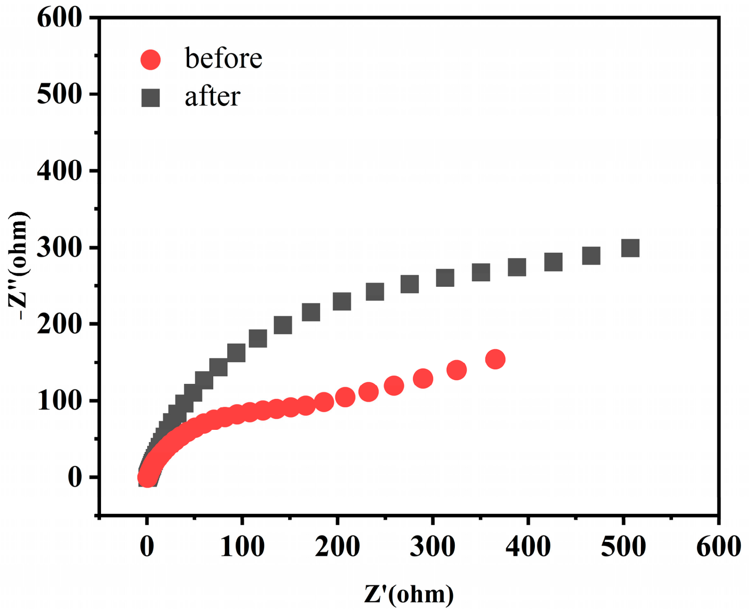

Figure S10. The EIS test of the Ni-MOF/$Ti_3C_2T_x$ before and after stability test.

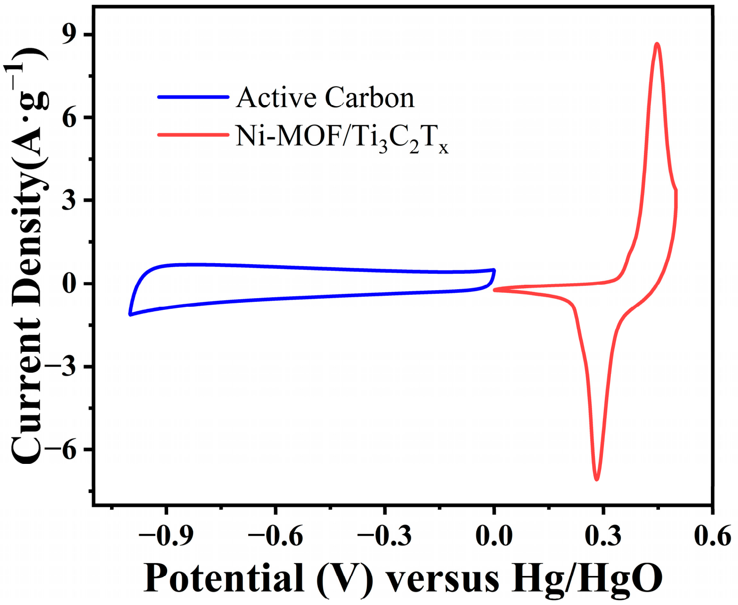

Figure S11. Energy density at different Power density of Ni-MOF/$Ti_3C_2T_x$.

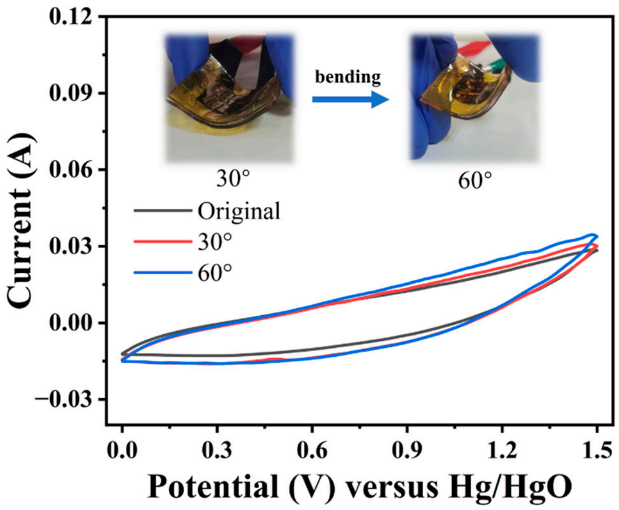

Figure S12. The CV test under original or bending states.
